# Rapid Scale-Up of Antiretroviral Treatment in Ethiopia: Successes and System-Wide Effects

**DOI:** 10.1371/journal.pmed.1000056

**Published:** 2009-04-28

**Authors:** Yibeltal Assefa, Degu Jerene, Sileshi Lulseged, Gorik Ooms, Wim Van Damme

**Affiliations:** 1 Health Programs Department, National HIV/AIDS Prevention and Control Office, Addis Ababa, Ethiopia; 2 International Center for AIDS Care and Treatment Programs, Columbia University, New York, New York, United States of America; 3 Department of Public Health, Institute of Tropical Medicine, Antwerp, Belgium

## Abstract

Yibeltal Assefa and colleagues describe the successes and challenges of the scale-up of antiretroviral treatment across Ethiopia, including its impact on other health programs and the country's human resources for health.

**Summary Points**

- There has been substantial expansion of access to ART and HIV counseling and testing in Ethiopia, whilst maintaining the performance of other health programs such as tuberculosis and maternal and child health services.
- Task shifting to the health officers, nurses, and health extension workers is thought to be responsible for these successes.
- However, HIV prevention interventions and management of chronic care patients are lagging behind. This may be due to lack of attention to these health care areas and to physicians leaving the public sector for NGOs, including AIDS-related NGOs.
- Prevention of HIV infection, retention of patients in chronic care, and retention of physicians in the public sector need urgent attention for effective and sustainable HIV/AIDS and health systems responses in the long term.

## Introduction

The provision of antiretroviral treatment (ART) has decreased morbidity and mortality in people living with HIV/AIDS [1],[2]. However, introducing ART to sub-Saharan Africa was a topic of hot debate just a few years ago. Concerns about adherence and subsequent development of drug resistance, poor infrastructure, logistic and human capacity, and cost-effectiveness were the major issues [3]. Once pilot projects indicated the feasibility of ART delivery in resource-limited settings, the World Health Organization catalyzed the global effort by declaring lack of access to effective HIV treatment a global emergency. This resulted in the “3 by 5” initiative, which aimed to provide 3 million people in developing countries with ART by the end of 2005 [4]—a 10-fold increase in two years, as in 2003, only 100,000 people living with HIV/AIDS in developing countries were able to access ART [4].

Since the declaration of the “3 by 5” initiative, there have been several enabling factors for the rapid and massive scale-up of ART in resource-limited settings. Some of these are: (1) increased funding, mainly through global initiatives such as the Global Fund To Fight AIDS, Tuberculosis and Malaria and the United States President's Emergency Plan for AIDS Relief (PEPFAR) [5]–[7]; (2) a dramatic reduction in prices of antiretroviral drugs through considerable negotiation [7]; and (3) the public health approach to ART delivery [8].

Although the “3 by 5” target was not achieved (only 1.3 million people were able to access ART by the end of 2005) [9], this ambitious plan has served several purposes, including revealing the real challenges for ART scale-up in developing countries [10]. Several developing countries have adopted the public health approach to ART delivery to increase access to treatment [8]. Experiences in ART scale-up using the World Health Organization's public health approach have been documented in countries with huge geographic, sociocultural, and demographic variations [11]. However, there have been few reports on the system-wide effects of rapid ART scale-up on the overall health system and the HIV epidemic within individual countries.

In this paper, we present Ethiopia's experience in expanding access to ART using the public health approach and highlight the system-wide effects associated with the rapid scale-up of the ART program. Our analysis is based on secondary data sources, mainly (1) official documents from the Ethiopian Ministry of Health, including annual health service coverage reports on health and health-related indicators such as HIV/AIDS, tuberculosis, maternal and child health, and human resources for health (HRH); (2) the national ART treatment and implementation guidelines and proceedings of HIV/AIDS program review meetings; and (3) monthly ART reports available on the National AIDS Resource Centre Web site (http://www.etharc.org/). Information gathered through interviews from key informants in the national and regional HIV/AIDS programs was also used to complement the data from secondary sources.

## The Antiretroviral Treatment Program in Ethiopia

According to the most recent estimates, about 1 million people (2.2% of the adult population) were living with HIV in Ethiopia in 2008. In the same year, approximately 290,000 people needed ART [12]. To respond to the treatment needs of people living with HIV/AIDS, the National Antiretroviral Drugs Policy was developed in 2002, and the first treatment guideline for adults and adolescents was issued in 2003 and revised in 2007 [13]. A fee-based ART program was officially started in 2003. Moreover, a number of initiatives have been undertaken to expand the availability of ART in Ethiopia, including those by the Global Fund, PEPFAR, the Ethiopian North American Health Professionals Association, the Clinton Foundation, and the Ethiopian Red Cross Society [5]. As a result, a free ART program was launched in early 2005. Under the guidance of the strategic plan for the multi-sectoral response, 2004–2008 [14] and the road map for accelerated access to ART, 2004–2006 and 2007–2008/10 [15],[16], the ART roll-out plan has been implemented. Consequently, ART services have been decentralized and have been available in both health centers and hospitals since August 2006 [17],[18].

## Rapid Expansion of HIV/AIDS Services: A Success Story (Except for HIV Prevention)

**Figure 1:**
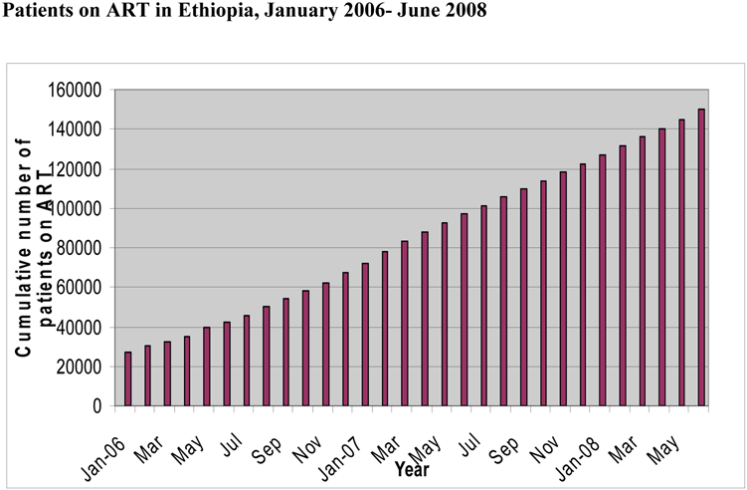
Patients on ART in Ethiopia, January 2006–June 2008.

The number of patients ever started on ART increased from 900 at the beginning of 2005 to more than 150,000 by June 2008 (Figure 1); and the number of patients enrolled for ART has also increased from 2,700 to 5,000 per month. The proportion of women and children, out of the total number of patients who received ART, increased from 25% in 2005 to 55% in 2008; the proportion of patients receiving ART outside Addis Ababa increased from 35% in 2005 to 75% in 2008 [18]. This has happened following the establishment of the decentralized and free ART program in the country since 2005. The number of clients receiving HIV counseling and testing services has also increased considerably, from 448,000 (between mid-2004 and mid-2005) to more than 4.5 million (between mid-2007 and mid-2008) [18].

The proportion of patients receiving chronic HIV care and ART (including newly enrolled patients and patients at follow-up consultations) in health centers increased almost 10-fold between October 2006 and October 2007, up from 2.8% to 17% and from 1.1% to 11.4%, respectively (Table 1).

**Table 1:** Decentralization of ART and chronic HIV care in Ethiopia, October 2006 and October 2007.

Type of Facilities	October 2006		October 2007	
	Chronic HIV Care	ART	Chronic HIV Care	ART
Health center	2,386 (2.8%)	570 (1.1%)	31,197 (17%)	12,496 (11.4%)
Hospital	83,975 (97.2%)	53,319 (98.9%)	156,573 (83%)	97,056 (88.6%)
Total	88,361 (100%)	53,889 (100%)	187,770 (100%)	109,552 (100%)

Source: Monthly ART reports, National HIV/AIDS Prevention and Control Office, Ethiopian Ministry of Health.

In spite of the above achievements, there is a widely held concern that HIV prevention activities have lacked focus and due attention. For example, it was estimated that there were 75,000 HIV-positive pregnant women in 2007, of whom only 8,000 (10.7%) received antiretroviral prophylaxis; in contrast, ART coverage was 48% in the same year [18]. Key informant interviews with national and regional HIV/AIDS program managers in 2006 and 2007 indicated that sexually transmitted infection prevention and treatment is somewhat neglected, which they associated with the intense focus on ART. The informants further stated that there is no single partner organization supporting the sexually transmitted infection prevention and treatment program in the country, while there are multiple partner organizations for AIDS care and treatment services. The percentage of women who knew that a healthy-looking person could have HIV increased only from 51% in 2005 to 53.2% in 2007; while comprehensive knowledge of HIV/AIDS prevention and transmission among the adult population was very low (22%) in 2005, with no improvement in 2007 [19],[20]. National HIV/AIDS estimates, based on calibrated data from antenatal care surveillance surveys and demographic and health surveys, indicate that the estimated and projected number of new HIV infections has been increasing since 2004 and will continue to increase until 2010 [12]. We should, however, be cautious in the comparison and interpretation of these data as they have different study populations and sample sizes.

The routine data supplied to the national HIV/AIDS prevention and control office indicate that many patients have dropped out of chronic care. By the end of June 2008, there were only 110,611 patients (75%) who were alive and on ART out of the 150,136 patients who had been started on ART since 2003 [18]. This indicates the need for an intervention to reduce the drop-out rate due to either death or loss to follow-up. Key informants have stressed that quality of care, including retention of patients in care, should be given equal attention as expanding access to treatment.

## System-Wide Effects: Positive Except for Internal Brain-Drain

The trend among physicians seems alarming as the number of physicians in the public services appears to have decreased from 1,613 in 2003 to 1,037 in 2007, while the number in the private for-profit and NGO (nongovernmental organization) sectors has increased from 419 in 2003 to 769 in 2007 [21]. It has been highly worrying that the “internal brain-drain” is depleting HRH in the public sector. Key informants confirmed that many highly skilled physicians have left the public sector to work with NGOs, mainly AIDS programs, in training and mentorship activities for health workers in the public sector. Therefore, these physicians working in the AIDS-related NGOs have left the public sector in the sense that they are no longer paid by the government; otherwise, they are still supporting the public sector, but exclusively for AIDS programs. On the other hand, the number of mid-and low-level health workers such as health officers and health extension workers has been increasing. The number of health officers increased from 631 in 2003 to1,151 in 2007; the training of health extension workers started in 2003 and increased to 17,653 in 2007 [21].

The number of tuberculosis cases detected has been stable in spite of the decline in the number of physicians who are conventionally responsible for the diagnosis and treatment of tuberculosis (Table 2). The public health sector seems to have coped quite well with the “internal brain-drain” of physicians by engaging more and more health officers in the diagnosis and treatment of tuberculosis. Tasks have shifted from physicians to health officers in Ethiopia because of the critical shortage of physicians in the country. Table 2 also shows that the tuberculosis treatment success rate has been increasing in spite of the declining number of physicians. This is an indication that services delivered by health officers are not of lesser quality (here quality means treatment success rate) compared to physicians; and health officers can provide quality service in the Ethiopian setting where there is a critical shortage of physicians.

**Table 2:** Trend of health services coverage (%) in Ethiopia, 2003–2007.

Year	Number of New Tuberculosis Cases Detected^a^	TuberculosisTreatment Success Rate^b^	EPI Coverage^c^	Contraceptive Acceptance Rate^d^	Antenatal Care Coverage^e^
**2003**	37,004	81	50.4	21.5	27.4
**2005**	38,525	81	70.1	25.2	42.1
**2007**	37,645	85	72.6	33.6	52.1

a The number of tuberculosis cases diagnosed during the reporting period.

b The proportion of tuberculosis patients who have completed their treatment and been cured.

c Expanded program of immunization: defined as receiving three doses of the combined vaccine against diphtheria, pertussis, and tetanus.

d The proportion of reproductive-age women (15–49 years) who are accepting a modern contraceptive method (new and repeat acceptors).

e The proportion of pregnant women who attended antenatal care at least once during their current pregnancy, and were seen by a health professional, for reasons related to their pregnancy.

Source: [21].

Between 2003 and 2007, immunization coverage, contraceptive acceptance rate, and antenatal care coverage all increased substantially (Table 2). These primary health care services are the tasks of nurses and health extension workers, whose numbers are increasing impressively in the country [21]. Physicians are less involved in the delivery of the primary health care services stated above; as a result, the number of physicians doesn't directly affect the primary health coverage in Ethiopia, given the adequate number of mid- and low-level health care workers.

We also analyzed mortality trends in under-fives, infants, and mothers. The under-five mortality rate had decreased greatly from 204 in 1990 to 123 in 2006; the infant mortality rate had also decreased from 122 in 1990 to 77 in 2006 [22]. This decline in mortality is due to the large number of health officers and nurses who are increasingly engaged in the delivery of clinical services to infants and children under five. The maternal mortality ratio also declined in the 1990–2006 period, although it remains very high at 673 per 100,000 live births. We think this is related to poor access to emergency obstetrics care [19] and the declining numbers of physicians in general health services, especially in the rural areas [21].

## The Way Forward

Access to ART has dramatically increased in Ethiopia over the last three years; this has been accompanied by an equally dramatic increase in the number of people tested for HIV, which has in turn enhanced access to care and treatment services. However, the number of patients dropping out of care is a concern that needs to be addressed with strategic interventions, including the chronic care model that links health care delivery with community- and home-based interventions.

It is also evident that the actual implementation of HIV prevention activities in the country is still lagging behind, and new HIV infections are still outpacing ART scale-up. It was indeed estimated that 125,000 new HIV infections occurred in 2007, but only 55,000 new patients were started on ART during the same year [12],[18]. We think the country needs to have a comprehensive approach, integrating prevention with the treatment scale-up program, for a sustainable HIV/AIDS response in the long term.

It is interesting to see that the performance of other health programs is not affected by the rapid expansion of the ART program, although it has contributed to some extent to the “internal brain-drain” of physicians. Nevertheless, the number of tuberculosis cases detected every year remained the same and treatment success rates improved. We attribute this to task shifting to health officers, who are replacing physicians on clinical tasks including tuberculosis diagnosis and treatment in the rural parts of the country. We thus encourage the further wide-scale utilization of these cadres, which are really becoming the backbone of the health system in the rural parts of the country. Moreover, access to many maternal and child health services has increased substantially thanks to the massive scale-up of the health extension program and the resultant overall increase in primary health care coverage since 2003 [19].

This analysis is the first of its kind trying to analyze and describe the system-wide effects of ART scale-up in Ethiopia. However, it is based on secondary data sources, which may lack accuracy and completeness. We also couldn't get the accurate number of physicians in the AIDS-related NGOs, assumed to “poach” the majority of physicians from the public sector.

We suggest the following areas for future in-depth research in Ethiopia: (1) the health extension program and the health officers program and their effect on health outcomes, (2) the contribution of AIDS-related NGOs in the HRH crisis, and (3) the effect of physician loss from the public sector on health outcomes in other health programs. We also recommend that the Ministry of Health improve data collection on health workforces in AIDS-related NGOs.

In conclusion, Ethiopia has been able to rapidly scale-up ART and HIV counseling and testing services with no evidence of decline in the performance of other health programs. However, prevention of HIV infection, retention of patients in chronic care, and retention of physicians in the public sector remain important challenges that need to be addressed urgently for a comprehensive and sustainable HIV/AIDS and health systems response.

## Abbreviations

ART: antiretroviral treatment
HRH: human resources for health
NGO: nongovernmental organization
PEPFAR: President's Emergency Plan for AIDS Relief
